# Draft genome sequence of carbapenems-resistant *Acinetobacter baumannii* Hakim RU_CBWP strain isolated from a pond surface water in Bangladesh

**DOI:** 10.1128/mra.00440-24

**Published:** 2024-06-12

**Authors:** Muhib Ullah Khan, M. Romance, Md. Arif-Uz-Zaman Polash, Nusrat Zahan, Md. Sumon Ali, Jafor Raihan, Subir Sarker, Md. Hakimul Haque

**Affiliations:** 1 Department of Veterinary and Animal Sciences, University of Rajshahi, Rajshahi, Bangladesh; 2 Biomedical Sciences & Molecular Biology, College of Public Health, Medical and Veterinary Sciences, James Cook University, Townsville, Queensland, Australia; Queens College Department of Biology, Queens, New York, USA

**Keywords:** whole genome, pond surface water, carbapenems resistant, *Acinetobacter baumannii*, Bangladesh

## Abstract

We have revealed the genomic sequence of *Acinetobacter baumannii* strain Hakim RU_CBWP isolated from pond surface water. Our assembled genome covers 3.787 Mb with 45.5629× coverage, showcasing an average GC content of 38.60%. This genome contains two CRISPR arrays, 17 prophages, 22 antibiotic resistance genes, and 20 virulence factor genes.

## ANNOUNCEMENT

As an opportunistic and ESKAPE pathogen, *Acinetobacter baumannii* poses a significant nosocomial infection risk (1). Its propensity to develop resistance to last-resort antibiotics, including colistin, tigecycline, and carbapenems, raises grave public health concerns (2). Transferring resistant genes via mobile genetic elements complicates treatment strategies, particularly for critically ill and immunocompromised patients (3, 4). Multidrug-resistant *Acinetobacter baumannii* was noted in humans, animals, food, and the environment worldwide, underscoring the crucial need for continuous surveillance and monitoring through One Health approaches to understanding its molecular epidemiology and implementing effective control measures (3, 5, 6).

The Institute of Biological Science (IBSc) at the University of Rajshahi, Bangladesh, approved all research techniques and protocols under Memo No. 56/321/IAMEBBC/IBSc. In September 2023, we collected pond surface water samples at the University of Rajshahi, Bangladesh, following standard procedures. The water samples were uniformly mixed, placed in sterile tubes, and transported to the laboratory (24.3733°N, 88.6049°E). We inoculated these samples on UTI agar (HiMedia, India) and incubated them aerobically at 37°C for 18–24 hours (7). *Acinetobacter baumannii* was isolated by streaking the cultures on MacConkey agar (HiMedia, India), followed by staining and biochemical tests (8). We performed antimicrobial susceptibility testing of the isolates using the disk diffusion method (9) following CLSI guidelines (10). The strain is resistant to amoxicillin, amoxicillin + clavulanic acid, cephradine, co-trimoxazole, azithromycin, and Gentamycin. We cultured the isolated strain in nutrient broth (HiMedia, India) overnight at 37°C and then extracted its genomic DNA using the Qiagen DNA Mini Kit (QIAGEN, Hilden, Germany). Genomic DNA underwent enzymatic fragmentation using the NEBNext dsDNA Fragmentase Kit (NEB, MA, USA), followed by size selection with SPRI beads (11). A sequencing library was prepared by the Nextera DNA Flex Library Preparation Kit (Illumina, San Diego, CA, USA), and the library was sequenced with 2 × 150 paired-end reads on the Illumina NextSeq2000 platform. Raw paired-end reads (*n* = 5,168,921) were trimmed with Trimmomatic.v0.39 (12), and genome assembly was conducted using Unicycler.v0.4.9 (13). Quality checks were performed using FastQC v0.11.7 (14), and annotation was carried out using PGAP v3.0 (15). The assembled genome was analyzed for antibiotic resistance genes (ARGs) using CARD v.3.2.4 with RGI v6.0.2 (16) and ResFinder v.4.1 (17), mobile genetic elements (MGEs) using mobileOG-db (18), virulence factor genes using VFDB with VFanalyzer (19), pathogenicity index using PathogenFinder v.1.1 (20), sequence type using MLST v.2.0 (21), CRISPR arrays using CRISPRimmunity (22), prophages using PHASTER (23), and metabolic functional features using RAST v.2.0 (24). Unless otherwise specified, we used the default parameters for all tools.

**Fig 1:**
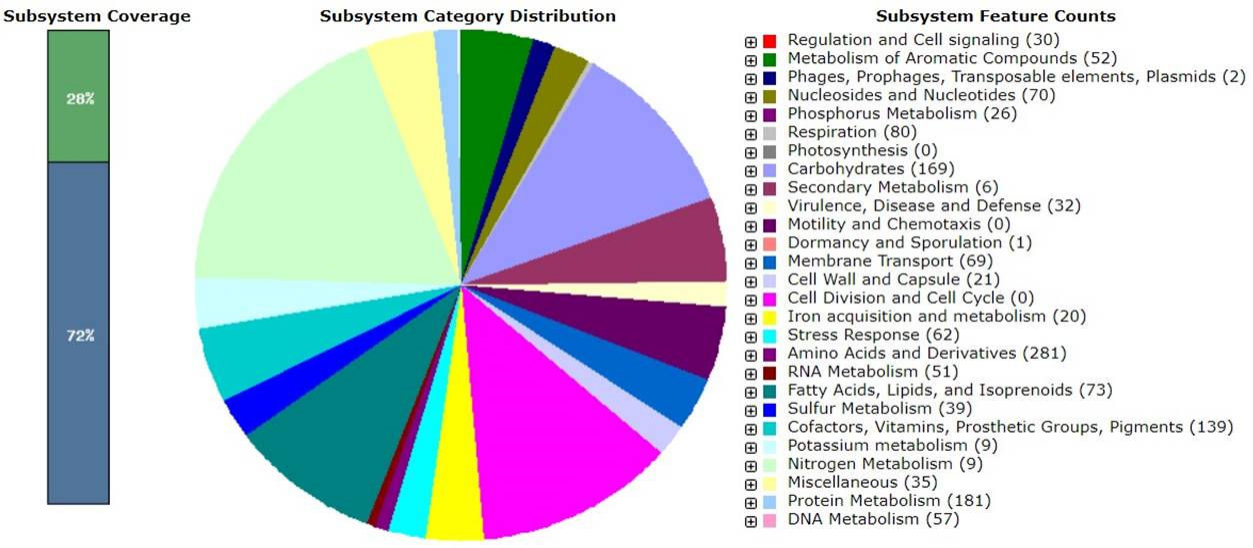
Metabolic functional features in the assembled genome of the *A. baumannii* Hakim RU_CBWP strain in SEED viewer.

The attributes of the draft genomes are noted in Table 1. Notably, 22 ARGs, 20 virulence genes, and 69 MGEs were identified. MLST classified the genome as sequence type unknown but nearest to 2168, 2133, 1938, 1379, 1422, 1452, 1447, 619, 2146, 2554, 1526, 1459, 25, 1450, 2145, 2831, and 1456, according to the PathogenFinder tool, which indicated a pathogenicity index of 0.861. The genome exhibited two CRISPR arrays with signature genes (*DEDDh*, *TnsC*, *WYL*, *cas3*, *TniQ*, *csa3*, and *cas3f*) and 17 prophages. RAST analysis uncovered 304 subsystems, comprising 3,679 genes with 28% coverage (Fig. 1).

**TABLE 1:** Genomic attributes of the *A. baumannii* strain Hakim RU_CBWP

Elements	Values
Genome size	3,787,050 bp
Genome coverage	45.5629×
G + C content	38.60%
Number of contigs	161
Contig L50	11
Contig N50	103,712 bp
Total genes	3,672
Coding sequences	3,621
Coding genes	3,554
RNA genes	51
tRNA genes	41
rRNAs genes	6
ncRNAs genes	4
Pseudo genes	67
Genes assigned to SEED subsystems	3,679
Number of subsystems	304

## Data Availability

The study on *Acinetobacter baumannii* Hakim RU_CBWP, conducted using the WGS shotgun approach, was submitted to NCBI/GenBank, and it was assigned the accession number JBCDME000000000. The pertinent data, including the original readings, were stored with BioProject accession number PRJNA1101523, BioSample accession number SAMN40996234, and SRA accession number SRR28724258. The specific version mentioned in this document is labeled as JBCDME000000000.1.
